# An Agreeable Surprise

**Published:** 1864-01

**Authors:** 


*An Agreeable Surprise.*—The Medical Class of the Buffalo University recently presented their Prof. of Anatomy, Dr. Eastman, with a valuable horse, as a testimonial of their "regard for him as a faithful friend—a thorough teacher—and christian physician." Although "taken entirely by surprise," the Doctor accepted the token in very befitting terms.

